# Bicruciate Reconstruction with Bilateral Hamstring Autografts: Technique and Functional Results

**DOI:** 10.1055/s-0043-1770970

**Published:** 2024-09-04

**Authors:** Bruno Aspirino Ciancio, Marina Mayumi Azuma, João Victor Medeiros De Cerqueira, Gustavo Kenzo Miyashita, Jorge Liozi Yamashita, Leonardo Addêo Ramos

**Affiliations:** 1 Hospital Nipo Brasileiro, São Paulo, SP, Brasil; 2 Departamento de Ortopedia e Traumatologia, Escola Paulista de Medicina, Universidade Federal de São Paulo, São Paulo, SP, Brasil

**Keywords:** anterior cruciate ligament, anterior cruciate ligament reconstruction, hamstring tendon, joint instability, knee dislocation, posterior cruciate ligament, posterior cruciate ligament reconstruction

## Abstract

**Objective** The purpose of this study was to evaluate the clinical and functional results of simultaneous reconstruction of the ACL and PCL with bilateral hamstring autografts. We hypothesized that this reconstruction technique results in less morbidity and has similar results to the ones published in the previous literature.

**Methods** Eighteen patients with bicruciate lesions were selected and treated by arthroscopic surgery with autologous hamstring tendons in a single-stage procedure. The thicker semitendinosus tendon (ST) and the two gracilis tendons (G) were used for a 6-strand PCL reconstruction. The thinner ST was used for a 3-strand ACL reconstruction. The average patient age at surgery was 31 years, and the minimum follow-up was 2 years. Function of the operated knee was evaluated according to the Lysholm scale. Anterior knee laxity was examined with a KT-1000 arthrometer. Posterior laxity was evaluated using stress radiographies.

**Results** Statistically significant improvements were found for all three measurements ( *p* < 0.001). Knee function by the Lysholm score increased from 43.8 ± 4.1 to 89.9 ± 3.8 post-surgery. The average anterior knee laxity improved from 5.2 + -0.8 mm initially to 2.4 + - 0.5 mm post-surgery. The posterior translation of the tibia relative to the femur decreased from 10 ± 3.4 mm to 3 ± 1.6 mm post-surgery. No patient showed loss of motion in extension or knee flexion.

**Conclusion** The simultaneous bicruciate reconstruction with bilateral hamstring autograft is a valuable option to achieve good functional outcomes and ligamentous stability.

## Introduction

The term “bicruciate” simultaneously refers to the anterior cruciate ligament (ACL) and posterior cruciate ligament (PCL) of the knee. Bicruciate lesions (BL) are rare and associated with knee dislocation. 1 Most of these injuries occur as a result of a high-energy mechanism, such o motor vehicle accidents and animal tramplings. 2 Knee dislocations can also occur as a result of a low-energy mechanism, such as hyperextension in a sports-related trauma.

Although the ACL has a poor biological potential for healing, the PCL has a more favorable environment for healing due to a well vascularized environment that allows to improve the regenerative process and restore its functional stability. 3 Other anatomical differences between the ACL and the PCL are morphological aspects, such as width and thickness between the intact PCL (13 mm) and ACL (10 mm), which can be associated with functional impairments after BL.

The best choice for treatment after BL remains unclear, but surgical procedures appear to provide more favorable clinical benefits. 4 Despite the availability of several operative options, some controversies remain unclear, such as time for surgery, single versus double-stage, open versus arthroscopic surgery, single versus double tunnel, and graft choice.

For simultaneous ACL and PCL reconstruction, different graft types may be considered, such as allografts, autografts, synthetics, or any combination thereof. There is no consensus about the best graft type. 5 6 However, autografts have been recommended for high-demand sports athletes and young patients. Meanwhile, the hamstring autograft has gained popularity for ACL reconstruction. The small diameter size of the hamstring autograft has been deemed a risk factor for early failure after ACL reconstruction. 7 One study with 20 patients with chronic ACL and PCL deficiency who underwent simultaneous single-stage arthroscopic reconstruction showed good knee joint function after the initial follow-up period at 24-month. 8

We conducted this study using the thinnest semitendinosus tendon (ST) for a 3-strand ACL graft and the two gracilis tendons (G) and the thickest ST for a 6-strand PCL graft to simultaneous ACL/PCL arthroscopic reconstruction. We prefer to use autografts because we do not have easy access to allografts. The purpose of this study was to evaluate the clinical and functional results of simultaneous reconstruction of the ACL and PCL with bilateral hamstring autografts. We hypothesized that this reconstruction technique results in less morbidity and has similar results to the ones published in the previous literature.

## Materials and Methods

This prospective study was conducted from May 2016 to May 2019 after approval by the ethics committee of our institution. During this period, there were 166 cases of multiligament knee injuries. Of these, eighteen patients were classified with complete bicruciate injury. After diagnosis, all participants used non-articulated braces for 6 weeks and underwent a rehabilitation protocol for at least 3 months for functional improvement and range of motion gain.

The diagnosis was made by an experienced single orthopedic surgeon knee specialist and confirmed with magnetic resonance by an experienced musculoskeletal radiologist. The preoperative outpatient evaluation consisted of instability tests 9 and X-rays under stress. For PCL injury, these X-rays were taken while the patient kneeled on the affected limb 10 to evaluate the posterior displacement of the tibia relative to the femur. We considered complete PCL injuries where the distance of the line drawn parallel from the posterior cortex of the tibia to the most posterior point of the Blumensaat line was greater than or equal to 8 mm. 11

The inclusion criteria for participants were: complete bicruciate injury, symptomatic functional instability. The exclusion criteria were: associated fractures, presence of symptomatic peripheral instability, confirmed by physical examination and stress X-rays, patients with flexion contracture >10 degrees and flexion <90 degrees, presence of angular deviation in the coronal plane, and presence of knee osteoarthritis greater than Ahlbäch grade 2. 12 All included patients agreed to participate and authorized the publication of surgery photographs, if necessary.

Patients were followed for at least 24 months. Knee function was assessed by the Lysholm scale and return to sports activities. Anterior stability was assessed using the KT-1000 arthrometer. Posterior translation was assessed by stress radiographs pre and with 24 months postoperative. Additionally, we evaluated the thickness graft, and complications related to the surgical procedure and postoperative.

Its important to note that when calibrating the KT-1000, we used the “ *step off”* of the contralateral knee joint line as a reference.

The procedure began by arthroscopy through the anteromedial (AM) and anterolateral (AL) portals. A leg holder was used, keeping the popliteal fossa free from compression. The surgeon confirmed complete bicruciate injury. The integrity of both collateral ligaments was confirmed through dynamic maneuvers.

**Fig. 1:**
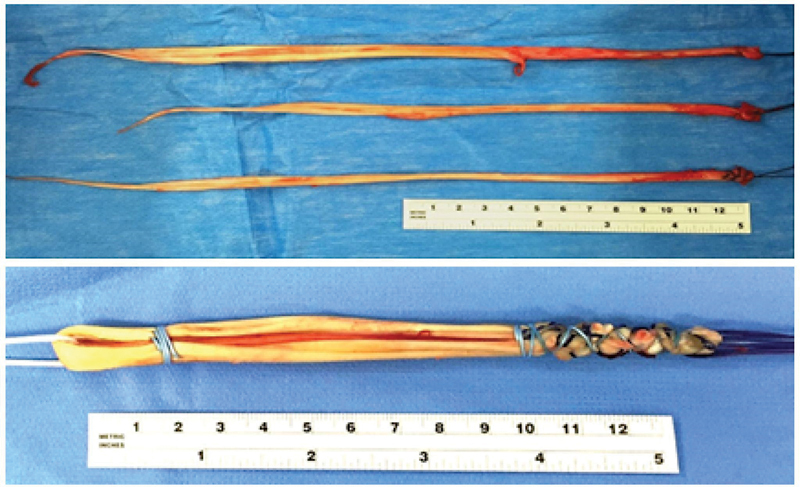
The 6-strand PCL graft with the thicker semitendinosus tendon and two gracilis tendons.

**Fig. 2:**
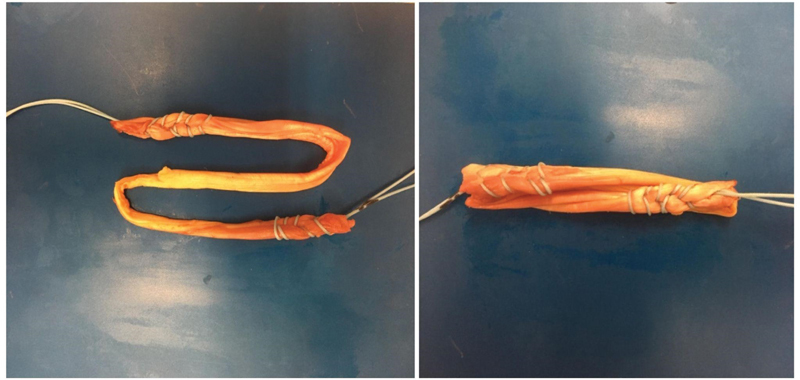
The 3-strand ACL graft with a thinner semitendinosus tendon.

After the confirmed diagnosis, the surgeon continued with the hamstring harvest of the ST and G bilaterally. To make a thicker PCL than the ACLThe two grafts were prepared as follows: After measured, the thicker ST tendon and the two G tendons were separated and doubled up once, yielding a 6-strand graft for PCL reconstruction ( Fig. 1 ); the remaining ST was folded 2 times onto itself, leaving a 3-strand graft for ACL reconstruction ( Fig. 2 ).

The arthroscopy continued by treating possible chondral and meniscal injuries. The ACL and PCL anatomical femoral points were determined at their respective femoral condyles and tibial origins. 13 14 Whenever possible, the original footprints were used for the tunnel position. With the camera through the AM portal in the intercondylar background under direct visualization, the posteromedial (PM) portal was made 2 cm above the joint line. With this technique, we captured a better view of the posterior aspect of the proximal tibia.

The surgeon drilled the PCL and ACL tunnels backward so that the leakage of saline solution through them did not obscure the intra-articular view due to the pressure decrease.

With the camera through the PM portal and the PCL tibial guide through the AM portal, the tibial tunnel was drilled using the Flipcutter® (Arthrex), which provided greater security of the neurovascular structures at the popliteal fossa and eliminated the need of drilling a larger diameter or the chance of guidewire migration at the time of drilling.

The camera was exchanged for the AL portal to drill the femoral ACL tunnel through the AM portal. To avoid a very short tunnel (<30mm) the knee was bent to 110 degrees at the time of drilling. We again exchanged the portals to drill the femoral PCL tunnel through the AL portal.

The surgeon then proceeded to the tibial ACL tunnel.

When the tunnels were ready, the PCL graft was first raised up and fixed with a bio-interference screw (BIS) at the femur. Then, the ACL graft was raised up and fixed with a RetroButton® (Arthrex) at the femur.

The final fixation of each graft at the tibia was performed with a BIS: first the PCL with the knee at 70 degrees of flexion and then the ACL with 20 degrees of flexion. Before fixing the screws, with the help of the thumb on the joint line, we checked and maintained the knee reduction.

All participants were prescribed the same postoperative management: functional immobilization for 6 weeks. Complete range of motion gain is recommended after this period. Partial weight bearing is started after four weeks. The rehabilitation protocol is started in the second week after surgery and continued for at least 8 months.

At 24 months post-surgery, the functional outcomes were assessed by the Lysholm Knee Scoring Scale, 15 the KT-1000 arthrometer, and the stress X-ray. 16

Statistical analysis was performed by the paired *t* -test. The significance level of 0.05 was adopted in all statistical tests, and the statistical program SPSS version 18.0 was used for all statistical analyses.

## Results

The average age of these 18 patients was 31 years (range: 17–49), and 17 were men. Regarding the mechanism of injury, 12 patients were injured in car acidentes, and 6 in sports practice. Four reported to be sedentary, while 14 practiced regular physical activity at different levels and modalities ( Table 1 ). Two patients (11%) lost the knee bending motion and one (5.5%) presented anterior knee pain. There were no infection complications. The mean thicknesses of the grafts for the ACL and PCL were 7.55 and 8.5 mm, respectively ( Table 1 ). The statistical analysis suggested significant improvements for the following variables: the Lysholm score ( Table 2 ), posterior displacement of the tibia through stress X-rays ( Table 3 ), and use of the KT-1000 arthrometer ( Table 4 ).

**Table 1:** Individual outcomes of each study participant

Patient number	Age (y)	Trauma	Sport	Lysholm score		KT-1000 arthrometer (mm)		Stress X-ray (mm)		Graft thickness (mm)	
				Pre	Post	Pre	Post	Pre	Post	ACL	PCL
01	36	E	Soccer	44	90	6	2.8	8	3	8	9
02	28	E	Soccer	44	90	4	2	10	3	7	8
03	27	A	Motocross	40	96	5	3	20	4	8	8
04	19	E	Judo	52	90	6	2	8	2	7	8
05	47	A	−	42	88	6	3	14	5	8	9
06	35	A	−	44	86	4	2	8	6	7	8
07	29	A	Soccer	42	86	6	3	10	6	8	8
08	42	A	−	40	82	6	3	8	2	8	9
09	29	E	Soccer	38	90	5	2.5	14	5	7	9
10	23	A	Soccer	40	90	6	2	12	4	8	10
11	46	A	Running	38	98	5	2	8	2	7	7
12	25	E	Skate	52	90	4	2	8	2	8	9
13	19	A	Motocross	48	88	5	2	6	2	7	9
14	30	A	Soccer	44	90	5	2.5	8	1	7	8
15	32	A	Soccer	44	84	6	2	10	1	8	9
16	29	E	Soccer	48	90	6	2	12	2	7	8
17	35	A	Running	44	92	4	2	8	3	8	9
18	26	E	−	44	86	5	3	8	1	8	8

Abbreviations: A, automotive injury; E, sports injury.

**Table 2:** Changes in the Lysholm score from pre- to postoperative evaluation

Evaluation	Lysholm score				
	Average	d.p.	Median	Low	High
Pre	43.8	4.1	44	38	52
Post	89.2	3.8	90	82	98
Variation (post-pre)	45.4	6.0	45	38	60
Paired *t* -test	p < 0.001				

**Table 3:** Changes in the posterior displacement of the tibia on stress X-ray from pre- to postoperative evaluation

Evaluation	Posterior displacement of the tibia on X-ray (mm)				
	Average	d.p.	Median	Low	High
Pre	10.0	3.4	8.0	6	20
Post	3.0	1.6	2.5	1	6
Variation (post-pre)	−7.0	3.0	−6.5	−16	−2
Paired *t* -test	p < 0.001				

**Table 4:** Changes in anterior knee laxity by KT-1000 arthrometer from pre- to postoperative evaluation

Evaluation	Anterior knee laxity by arthrometer (mm)				
	Average	d.p.	Median	Low	High
Pre	5.2	0.8	5	4	6
Post	2.4	0.5	2	2	3
Variation (post-pre)	−2.8	0.8	−3	−4	−2
Paired *t* -test	p < 0.001				

The Lysholm scale was used to analyze subjective symptoms. The mean preoperative Lysholm score was 43.8 ± 4.1 (range: 38–52). The mean postoperative Lysholm score was 89.9 ± 3.8 (range: 82–98). After the two-year follow-up, according to this scale, 16 of 18 (88%) patients displayed excellent results, and 2 (12%) patients displayed good results. A significant improvement in the Lysholm score from preoperative to the final follow-up was observed ( *p* < 0.001) ( Table 2 ).

Stress X-rays were used to analyze PCL function. The mean preoperative value was 10 ± 3.4 mm (range: 6–20). After the two-year follow-up, this value fell to 3 ± 1.6 mm (range: 1–6), showing a significant improvement in posterior tibial displacement ( Table 3 ).

Although the selected cases did not present symptomatic peripheral lesions, we observed values of tibial posteriorization on stress radiographs above 12 mm. We believe that the injury of other knee restrictors such as the posterior capsule and menisci may have contributed to these.

The KT-1000 arthrometer examination showed that the mean side-to-side difference of anterior-posterior laxity was 5.2 ± 0.8 mm before surgery. After the two-year follow-up, this mean difference fell to 2.4 ± 0.5 mm ( Table 4 ).

Fourteen patients declared regular sports activity before their injury ( Table 1 ). After surgery, 8 (57%) were able to return to their sports activities. We did not consider their sports levels before and after surgery.

## Discussion

The most important finding in this study was the improvement in the functional outcomes after bicruciate reconstruction with bilateral hamstring autografts. Bicruciate lesions are rare and are associated with knee dislocation. Some studies have shown that primary surgical reconstruction yields better results than conservative treatment with regard to the objective stability and motion of the joints. Simultaneous ACL and PCL reconstruction through open procedures have also been reported with good results. 7 8 9 10 Previous techniques have used one or two stages with different graft types. 5 6

Literature has discussed the best time to operate as well as the number of procedures. The systematic review for multiple ligament knee injuries, Mook et al. 17 reported that acute surgery is highly associated with range of motion deficits and that staged procedures may produce better subjective outcomes and lower rates of range of motion deficits. Chuang et al. 18 described a rehabilitation protocol of a 3-stage program in which after 12 weeks the full range of motion was achieved. Then, the 1-stage reconstruction of the ACL and PCL was performed. A single-stage procedure was chosen for better stabilization of the knee, thus reducing the chance of ligament loosening.

The issue of single or double bundles are also discussed in ACL and PCL reconstruction. While the literature has shown some evidence that double-bundle have superior results in objective measures of knee stability, no results of clinical function have shown significant differences between both reconstructions. 19 In a recent systematic review, Shin et al. 20 found no clinically important differences between the transtibial and tibial inlay approach for PCL reconstruction. Fanelli et al. 21 reported their technique of a simultaneous arthroscopic reconstruction of the ACL and PCL in 1996 and the 2- to 10-year follow-up results in 2002. 22

La Prade et al. 23 demonstrated that single-staged multiple-ligament reconstruction with immediate post operative rehabilitation improved outcomes, including ROM, with low complication rates.

In 2006, Zhao et al. 24 published their ACL/PCL single-stage reconstruction technique with autogenous hamstring tendons. They used the ST tendon and the G tendon from the uninjured leg for a 6- to 8-strand PCL graft and the ST of the injured leg for a 4-strand ACL graft. Their results showed a KT-1000 side-to-side difference of anterior laxity at 25 degrees of flexion of 0 to 2 mm in 8 patients, 3 to 5 mm in 3 patients, and 7 mm in 1 patient. The Lysholm score post-surgery was 91.8 ± 4.6, similar to our present study. This is the closest technique to ours and had similar results. The main difference between them was in the preparation of the graft.

In 2015, Denti el al. 8 described a simultaneous arthroscopic reconstruction of BL comparing allograft tendons versus bone-patellar tibial-bone autograft for PCL and hamstring for ACL. They reported a mean Lysholm score of 93.9 ± 3.9 for the allograft and 89.1 ± 7.6 for the autograft. No significant strength deficit was found in both groups.

Inada and Piedade 25 published in 2021 a retrospective analysis of 25 patients treated by a two-stage technique. PCl inlay with patellar tendon and three months later an Acl reconstruction with hamstring tendons. They reported that 60% of the patients scored zero or + at the posterior drawer test, while 40% scored ++; 60% of patients were evaluated as good/excellent according to the Lysholm scale. Only one patient reached the pre-injury Tegner activity level. Injury duration had a negative influence on functional limitation.

For our technique, we chose the hamstring tendon due to less anterior knee pain, extension loss, and donor site morbidity. 26 By combining it with an extra gracilis tendon, we were able to enlarge the graft thickness for the PCL reconstruction. The maximum strength for a quadruple ST/G tendon was measured at ∼4.000 N in a study by Horner et al. 27 While a 4-strand graft appears to be sufficient for PCL reconstruction, it is our opinion that a 6-strand graft with an extra double gracilis, increasing graft thickness, produces a stronger graft.

As described in the papers above, different grafts bring similar results. The choice depends on the surgeon's preference. In knees with peripheral injuries, we choose the hamstrings for reconstruction of these and grafts of the extensor mechanism to the central pivot.

Moatshe et al. 28 conducted a biomechanical study in 2018 in which a better result was observed by first tensioning the PCL, then the ACL and then the posterolateral corner. Franciozi et al. 29 used a simultaneous tensioning protocol in BL. They found a better tibiofemoral orientation in the group that ACL fixation was made first. Based in our and others authors experiences 21 22 30 we prefer first to fix the PCL at 70 degrees of flexion, keeping the knee reduced and then the ACL with 20 degrees of flexion.

In a retrospective study of open complete single-stage reconstruction of complex ligament knee lesions, Hirschmann et al. 31 found that 79% of the patients were able to return to their previous sports practice, but only 33% reached their identical preinjury sport activity level. We believe that the different findings in our study are due to the lower level of athletes evaluated, assuming that elite athletes adhere to a more intensive rehabilitation protocol.

There were no infection. Two pacients had arhtrofibrosis and received manipulation 3 months after surgery. One patient had thrombosis despite the use of anticoagulant and was treated with a higher dose with a good outcome

The weaknesses of this study were the small number of patients, the absence of a control group, and the lack of a second observer to evaluate the results.

## Conclusion

The bicruciate reconstruction with bilateral hamstring autograft is a valuable option to achieve good functional outcomes and joint stability.
