# Supplementary material for: Bicruciate Reconstruction with Bilateral Hamstring Autografts: Technique and Functional Results
Source: Rev Bras Ortop (Sao Paulo). 2024 Sep 4;59(4):e542–8. doi: 10.1055/s-0043-1770970 (PMC11374398; doi:10.1055/s-0043-1770970)
Supplement: Related texts

# Research article: Reconstrução dos ligamentos cruzados anterior e posterior com autoenxertos bilaterais dos isquiotibiais: Técnica e resultados funcionais

**Keywords:** instabilidade articular, ligamento cruzado anterior, ligamento cruzado posterior, luxação do joelho, reconstrução do ligamento cruzado anterior, reconstrução do ligamento cruzado posterior, tendões dos músculos isquiotibiais

## Abstract

**Objetivo** O objetivo deste estudo foi avaliar os resultados clínicos e funcionais da reconstrução simultânea do ligamento cruzado anterior (LCA) e ligamento cruzado posterior (LCP) com autoenxertos bilaterais de isquiotibiais. Nossa hipótese é que esta técnica de reconstrução gera menor morbidade e tem resultados semelhantes aos publicados na literatura anterior.

**Métodos** Dezoito pacientes com lesões em LCA e LCP foram selecionados e tratados por cirurgia artroscópica com tendões isquiotibiais autólogos em procedimento único. O tendão semitendinoso (ST) mais espesso e os dois tendões do músculo grácil (G) foram usados para a reconstrução do LCP com seis fios. O ST mais fino foi usado para reconstrução do LCA com três fios. A média de idade dos pacientes à cirurgia foi de 31 anos e o acompanhamento mínimo foi de 2 anos. A função do joelho operado foi avaliada de acordo com a escala de Lysholm. A lassidão anterior do joelho foi examinada com um artrômetro KT-1000. A lassidão posterior foi determinada por meio de radiografias de estresse.

**Resultados** Melhoras estatisticamente significativas foram observadas nas três medidas ( *p* < 0,001). A função do joelho pelo escore de Lysholm aumentou de 43,8 ± 4,1 para 89,9 ± 3,8 após a cirurgia. A lassidão anterior média do joelho melhorou de 5,2 ± 0,8 mm para 2,4 ± 0,5 mm após a cirurgia. A translação posterior da tíbia em relação ao fêmur diminuiu de 10 ± 3,4 mm para 3 ± 1,6 mm no período pós-operatório. Nenhum paciente apresentou perda de movimento em extensão ou flexão do joelho.

**Conclusão** A reconstrução simultânea do LCA e do LCP com autoenxerto bilateral dos isquiotibiais é uma opção valiosa para obtenção de bons resultados funcionais e estabilidade ligamentar.

## Introdução

Os ligamentos cruzados do joelho são o ligamento cruzado anterior (LCA) e o ligamento cruzado posterior (LCP). As lesões dos dois ligamentos, chamadas de lesões bicruzadas (LB), são raras e associadas à luxação do joelho. 1 A maioria dessas lesões é causada por mecanismos de alta energia, como acidentes automobilísticos e atropelamentos envolvendo animais. 2 As luxações do joelho também podem ser provocadas por mecanismos de baixa energia, como a hiperextensão em um trauma esportivo.

Embora o LCA tenha baixo potencial biológico de cicatrização, o LCP apresenta ambiente mais favorável à cicatrização por ser bem vascularizado, o que permite melhorar o processo regenerativo e restaurar sua estabilidade funcional. 3 Outras diferenças anatômicas entre o LCA e o LCP são aspectos morfológicos, como a largura e a espessura entre o LCP (13 mm) e o LCA (10 mm) íntegros, que podem estar associados a déficits funcionais após a LB.

A melhor escolha para o tratamento da LB ainda é incerta, mas os procedimentos cirúrgicos parecem proporcionar benefícios clínicos mais favoráveis. 4 Apesar da disponibilidade de várias opções cirúrgicas, há algumas controvérsias, como tempo até a cirurgia, realização em estágio único ou duplo, procedimento aberto ou artroscópico, túnel único ou duplo e a escolha do enxerto.

Diferentes tipos de enxerto podem ser considerados para a reconstrução simultânea do LCA e do LCP, como aloenxertos, autoenxertos, enxertos sintéticos ou qualquer combinação desses elementos. Não há consenso sobre o melhor tipo de enxerto. 5 6 No entanto, os autoenxertos têm sido recomendados para atletas de alta demanda e pacientes jovens. Enquanto isso, o autoenxerto de isquiotibiais ganhou popularidade para a reconstrução do LCA. O pequeno diâmetro do autoenxerto de isquiotibiais foi considerado um fator de risco para falência precoce após a reconstrução do LCA. 7 Um estudo com 20 pacientes com deficiência crônica de LCA e LCP submetidos à reconstrução artroscópica simultânea em estágio único revelou boa função da articulação do joelho após o período inicial de acompanhamento de 24 meses. 8

Conduzimos este estudo usando o tendão semitendinoso (ST) mais delgado para enxerto de LCA com três fios e os dois tendões do músculo grácil (G) e o ST mais espesso para enxerto de LCP com seis fios para reconstrução artroscópica simultânea de LCA e LCP. Preferimos usar autoenxertos devido à ausência de fácil acesso a aloenxertos. O objetivo deste estudo foi avaliar os resultados clínicos e funcionais da reconstrução simultânea do LCA e LCP com autoenxertos bilaterais de isquiotibiais. Nossa hipótese é que esta técnica de reconstrução gera menor morbidade e tem resultados semelhantes aos publicados na literatura anterior.

## Materiais e Métodos

Este estudo prospectivo foi realizado de maio de 2016 a maio de 2019 após aprovação do comitê de ética de nossa instituição. Nesse período, houve 166 casos de lesões multiligamentares do joelho. Destes, dezoito pacientes foram classificados com lesão bicruzada completa. Após o diagnóstico, todos os participantes usaram órteses não articuladas por 6 semanas e foram submetidos a um protocolo de reabilitação por pelo menos 3 meses para melhora funcional e ganho de amplitude de movimento.

O diagnóstico foi feito por um único cirurgião ortopédico experiente, especialista em joelho, e confirmado por ressonância magnética por um radiologista musculoesquelético também experiente. A avaliação ambulatorial pré-operatória consistiu em testes de instabilidade 9 e radiografias de estresse. Nas lesões de LCP, essas radiografias foram obtidas com o paciente ajoelhado sobre o membro acometido 10 para avaliação do deslocamento posterior da tíbia em relação ao fêmur. As lesões do LCP foram consideradas completas caso a distância da linha traçada paralelamente da cortical posterior da tíbia ao ponto mais posterior da linha de Blumensaat fosse maior ou igual a 8 mm. 11

Os critérios de inclusão dos participantes foram lesão bicruzada completa e instabilidade funcional sintomática. Os critérios de exclusão foram fraturas associadas, presença de instabilidade periférica sintomática (confirmada por exame físico e radiografia de estresse), pacientes com contratura em flexão >10 graus e flexão <90 graus, presença de desvio angular no plano coronal e presença de osteoartrite do joelho com grau de Ahlbäch acima de 2. 12 Todos os pacientes incluídos concordaram em participar e autorizaram a publicação de fotografias cirúrgicas, caso necessário.

Os pacientes foram acompanhados por pelo menos 24 meses. A função do joelho foi avaliada pelo escore de Lysholm e o retorno às atividades esportivas. A estabilidade anterior foi analisada com artrômetro KT-1000. A translação posterior foi avaliada por radiografias de estresse antes e 24 meses após a cirurgia. Além disso, avaliamos a espessura do enxerto e as complicações relacionadas ao procedimento cirúrgico e pós-operatórias.

É importante notar que, ao calibrar o KT-1000, usamos o “ *step off”* da linha do joelho contralateral como referência.

O procedimento começou com a artroscopia pelos portais anteromedial (AM) e anterolateral (AL). Um suporte de membro inferior foi usado para manter a fossa poplítea livre de compressão. O cirurgião confirmou a presença de lesão bicruzada completa. A integridade de ambos os ligamentos colaterais foi confirmada por meio de manobras dinâmicas.

Após a confirmação do diagnóstico, o cirurgião procedeu à coleta isquiotibial do ST e G bilateralmente. Para fazer um LCP mais espesso que o LCA, os dois enxertos foram preparados da seguinte forma: depois da medida, o tendão ST mais espesso e os dois tendões G foram separados e dobrados uma vez, formando um enxerto de seis fios para reconstrução do LCP ( Fig. 1 ); o ST remanescente foi dobrado duas vezes sobre si mesmo, deixando um enxerto de três fios para reconstrução do LCA ( Fig. 2 ).

**Fig. 1:** Enxerto de ligamento cruzado posterior com seis fios e tendão semitendinoso mais espesso e dois tendões do músculo grácil.

**Fig. 2:** Enxerto de ligamento cruzado com três fios e tendão semitendinoso mais delgado.

A artroscopia prosseguiu para tratamento de possíveis lesões condrais e meniscais. Os pontos femorais anatômicos do LCA e LCP foram determinados em seus respectivos côndilos femorais e origens tibiais. 13 14 Sempre que possível, os pontos originais foram usados para posicionamento do túnel. Com a câmera colocada no portal AM, no fundo intercondilar e sob visualização direta, o portal posteromedial (PM) foi feito 2 cm acima da linha articular. Esta técnica permite a melhor visão do aspecto posterior da tíbia proximal.

O cirurgião perfurou os túneis do LCP e do LCA para trás para que o vazamento de soro fisiológico por eles não obstruísse a visão intra-articular por diminuição da pressão.

Com a câmera pelo portal PM e o guia tibial do LCP pelo portal AM, o túnel tibial foi perfurado com Flipcutter® (Arthrex), o que proporcionou maior segurança para as estruturas neurovasculares na fossa poplítea e eliminou a necessidade de perfuração de um maior diâmetro ou a possibilidade de migração do fio-guia no momento desse procedimento.

A câmera foi colocada no portal AL para perfuração do túnel femoral do LCA pelo portal AM. Para evitar que o túnel fosse muito curto (<30 mm), o joelho foi dobrado a 110 graus no momento da perfuração. Os portais foram novamente trocados para perfuração do túnel femoral do LCP pelo portal AL.

O cirurgião, então, procedeu ao túnel tibial do LCA.

Com os túneis prontos, o enxerto do LCP foi inicialmente elevado e fixado com parafuso de biointerferência (BIS) no fêmur. Em seguida, o enxerto do LCA foi elevado e fixado com RetroButton® (Arthrex) no fêmur.

A fixação final de cada enxerto na tíbia foi feita com um BIS: primeiro, no LCP, com o joelho a 70 graus de flexão e, depois, no LCA, com 20 graus de flexão. Antes da fixação dos parafusos, com o auxílio do polegar na linha articular, a redução do joelho foi verificada e mantida.

Todos os participantes foram submetidos ao mesmo manejo pós-operatório, ou seja, imobilização funcional por 6 semanas. Depois desse período, recomendou-se o ganho completo de amplitude de movimento. A carga parcial de peso começou após quatro semanas. O protocolo de reabilitação foi iniciado na segunda semana após a cirurgia e mantido por pelo menos 8 meses.

Aos 24 meses após a cirurgia, os resultados funcionais foram avaliados pela Escala de Pontuação do Joelho de Lysholm, 15 o artrômetro KT-1000 e radiografias de estresse. 16

A análise estatística foi realizada pelo teste *t* pareado. O nível de significância de 0,05 foi adotado em todos os testes estatísticos. Todas as análises foram realizadas no *software* SPSS versão 18.0.

## Resultados

A idade média desses 18 pacientes foi de 31 anos (variação: 17-49); 17 indivíduos eram do sexo masculino. Em relação ao mecanismo da lesão, 12 pacientes sofreram lesões em acidentes automobilísticos e seis, em práticas esportivas. Quatro relataram ser sedentários, enquanto 14 praticavam atividade física regular em diferentes níveis e modalidades ( Tabela 1 ). Dois pacientes (11%) perderam o movimento de flexão do joelho e um (5,5%) indivíduo apresentou dor anterior do joelho. Não houve complicações infecciosas. As espessuras médias dos enxertos de LCA e LCP foram de 7,55 e 8,5 mm, respectivamente ( Tabela 1 ). A análise estatística sugeriu melhoras significativas das seguintes variáveis: o escore de Lysholm ( Tabela 2 ), o deslocamento posterior da tíbia em radiografias de estresse ( Tabela 3 ) e a medida com artrômetro KT-1000 ( Tabela 4 ).

**Tabela 1:** Resultados individuais de cada participante do estudo

Número do paciente	Idade (anos)	Trauma	Esporte	Escore de Lysholm		Artrômetro KT-1000 (mm)		Radiografia de estresse (mm)		Espessura do enxerto (mm)	
				Pré	Pós	Pré	Pós	Pré	Pós	LCA	LCP
01	36	E	Futebol	44	90	6	2,8	8	3	8	9
02	28	E	Futebol	44	90	4	2	10	3	7	8
03	27	A	Motocross	40	96	5	3	20	4	8	8
04	19	E	Judô	52	90	6	2	8	2	7	8
05	47	A	−	42	88	6	3	14	5	8	9
06	35	A	−	44	86	4	2	8	6	7	8
07	29	A	Futebol	42	86	6	3	10	6	8	8
08	42	A	−	40	82	6	3	8	2	8	9
09	29	E	Futebol	38	90	5	2,5	14	5	7	9
10	23	A	Futebol	40	90	6	2	12	4	8	10
11	46	A	Corrida	38	98	5	2	8	2	7	7
12	25	E	Skate	52	90	4	2	8	2	8	9
13	19	A	Motocross	48	88	5	2	6	2	7	9
14	30	A	Futebol	44	90	5	2,5	8	1	7	8
15	32	A	Futebol	44	84	6	2	10	1	8	9
16	29	E	Futebol	48	90	6	2	12	2	7	8
17	35	A	Corrida	44	92	4	2	8	3	8	9
18	26	E	−	44	86	5	3	8	1	8	8

Abreviaturas: A, lesão automotiva; E, lesão esportiva; LCA, ligamento cruzado anterior; LCP, ligamento cruzado posterior; pré, pré-operatório; pós, pós-operatório.

**Tabela 2:** Alterações no escore de Lysholm entre as avaliações pré-operatória e pós-operatória

Avaliação	Escore de Lysholm				
	Média	Desvio-padrão	Mediana	Mínimo	Máximo
Pré-operatória	43,8	4,1	44	38	52
Pós-operatória	89,2	3,8	90	82	98
Variação (pós-operatória-pré-operatória)	45,4	6,0	45	38	60
Teste *t* pareado	p < 0,001				

**Tabela 3:** Alterações no deslocamento posterior da tíbia à radiografia de estresse entre as avaliações pré-operatória e pós-operatória

Avaliação	Deslocamento posterior da tíbia à radiografia (mm)				
	Média	Desvio-padrão	Mediana	Mínimo	Máximo
Pré-operatória	10,0	3,4	8,0	6	20
Pós-operatória	3,0	1,6	2,5	1	6
Variação (pós-operatória-pré-operatória)	−7,0	3,0	−6,5	−16	−2
Teste *t* pareado	p < 0,001				

**Tabela 4:** Alterações na lassidão anterior do joelho medida por artrômetro KT-1000 entre as avaliações pré-operatória e pós-operatória

Avaliação	Lassidão anterior do joelho medida com artrômetro (mm)				
	Média	Desvio-padrão	Mediana	Mínimo	Máximo
Pré-operatória	5,2	0,8	5	4	6
Pós-operatória	2,4	0,5	2	2	3
Variação (pós-operatória-pré-operatória)	−2,8	0,8	−3	−4	−2
Teste *t* pareado	p < 0,001				

A escala de Lysholm analisou sintomas subjetivos. A média do escore Lysholm pré-operatório foi de 43,8 ± 4,1 (intervalo: 38–52). O escore médio de Lysholm pós-operatório foi de 89,9 ± 3,8 (intervalo: 82–98). Após o acompanhamento por dois anos, de acordo com essa escala, 16 de 18 (88%) pacientes apresentaram resultados excelentes e dois (12%) pacientes tiveram bons resultados. Observou-se melhora significativa no escore de Lysholm desde o pré-operatório até o último acompanhamento ( *p* < 0,001) ( Tabela 2 ).

As radiografias de estresse avaliaram a função do LCP. O valor médio pré-operatório foi de 10 ± 3,4 mm (intervalo: 6–20). Após o acompanhamento de dois anos, esse valor caiu para 3 ± 1,6 mm (variação: 1–6), mostrando uma melhora significativa no deslocamento tibial posterior ( Tabela 3 ).

Embora os casos selecionados não apresentassem lesões periféricas sintomáticas, observamos valores de posteriorização tibial nas radiografias de estresse acima de 12 mm. Acreditamos que a lesão de outros restritores do joelho, como a cápsula posterior e os meniscos, possa ter contribuído para isso.

O exame com artrômetro KT-1000 mostrou que a diferença média da lassidão anteroposterior entre os dois lados foi de 5,2 ± 0,8 mm antes da cirurgia. Após dois anos de acompanhamento, essa diferença média caiu para 2,4 ± 0,5 mm ( Tabela 4 ).

Catorze pacientes declararam a realização regular de atividades esportivas antes da lesão ( Tabela 1 ). Após a cirurgia, oito (57%) conseguiram retornar às atividades esportivas. Não foram considerados os níveis esportivos antes e depois da cirurgia.

## Discussão

O achado mais importante neste estudo foi a melhora nos resultados funcionais após a reconstrução bicruzada com autoenxertos bilaterais de isquiotibiais. As lesões bicruzadas são raras e estão associadas à luxação do joelho. Alguns estudos mostram que a reconstrução cirúrgica primária tem resultados melhores do que o tratamento conservador no que diz respeito à estabilidade objetiva e ao movimento das articulações. A reconstrução simultânea do LCA e do LCP por meio de procedimentos abertos também foi relatada com bons resultados. 7 8 9 10 As técnicas anteriores usaram um ou dois estágios e diferentes tipos de enxertos. 5 6

A literatura discute o melhor momento da cirurgia, bem como o número de procedimentos. Em uma revisão sistemática de lesões ligamentares múltiplas do joelho, Mook et al. 17 relataram que a cirurgia aguda está altamente associada a déficits de amplitude de movimento e que procedimentos em estágios podem gerar melhores resultados subjetivos e taxas menores de déficits de amplitude de movimento. Chuang et al. 18 descreveram um protocolo de reabilitação em um programa de três estágios com alcance da amplitude total de movimento depois de 12 semanas. Em seguida, foi realizada a reconstrução em um estágio do LCA e do LCP. Optou-se por procedimento em tempo único para melhor estabilização do joelho, reduzindo, assim, a chance de soltura ligamentar.

A questão dos feixes simples ou duplos também é discutida na reconstrução do LCA e do LCP. Embora a literatura tenha mostrado algumas evidências de que o feixe duplo tem resultados superiores em medidas objetivas de estabilidade do joelho, nenhum achado de função clínica mostrou diferenças significativas entre as duas reconstruções. 19 Em uma revisão sistemática recente, Shin et al. 20 não observaram diferenças clinicamente importantes entre a abordagem direta ( *inlay* ) transtibial e tibial para reconstrução do LCP. Fanelli et al. 21 relataram sua técnica de reconstrução artroscópica simultânea do LCA e LCP em 1996 e os resultados de acompanhamento de 2 a 10 anos em 2002. 22

La Prade et al. 23 demonstraram que a reconstrução multiligamentar em estágio único, com reabilitação pós-operatória imediata, melhorou os resultados, inclusive a amplitude de movimento, com baixas taxas de complicações.

Em 2006, Zhao et al. 24 publicaram sua técnica de reconstrução em estágio único do LCA/LCP com tendões isquiotibiais autógenos. Estes autores utilizaram o tendão ST e o tendão G do membro inferior não acometido como enxerto de LCP de seis a oito fios e o ST do membro lesionado como enxerto de LCA de quatro fios. Seus resultados mostraram uma diferença entre os dois lados da medida de lassidão anterior por KT-1000 em 25 graus de flexão de 0 a 2 mm em oito pacientes, 3 a 5 mm em três pacientes e 7 mm em um paciente. O escore de Lysholm pós-operatório foi de 91,8 ± 4,6, semelhante ao observado em nosso estudo. Esta é a técnica mais próxima da nossa e teve resultados semelhantes. A principal diferença entre elas se referia ao preparo do enxerto.

Em 2015, Denti el al. 8 descreveram uma reconstrução artroscópica simultânea de LB comparando aloenxertos tendíneos e autoenxertos de osso-tibial patelar-osso em LCP e isquiotibiais em LCA. Esses autores relataram uma pontuação média de Lysholm de 93,9 ± 3,9 com o aloenxerto e 89,1 ± 7,6 com o autoenxerto. Não houve déficit significativo de força em nenhum dos grupos.

Inada e Piedade 25 publicaram, em 2021, uma análise retrospectiva de 25 pacientes tratados com uma técnica em dois estágios: *inlay* de LCP com tendão patelar e, três meses depois, reconstrução do LCA com tendões isquiotibiais. Estes autores relataram que 60% dos pacientes pontuaram zero ou + no teste de gaveta posterior, enquanto 40% pontuaram ++; 60% dos pacientes foram avaliados como bons/excelentes pela escala de Lysholm. Apenas um paciente atingiu o nível de atividade de Tegner pré-lesão. A duração da lesão teve influência negativa na limitação funcional.

Em nossa técnica, escolhemos o tendão isquiotibial devido à menor dor anterior no joelho, perda de extensão e morbidade no sítio doador. 26 Ao combiná-lo com um tendão grácil extra, conseguimos aumentar a espessura do enxerto para a reconstrução do LCP. A força máxima em um tendão ST/G quádruplo foi de cerca de 4.000 N em um estudo de Horner et al. 27 Enquanto um enxerto de quatro fios parece suficiente para a reconstrução do LCP, acreditamos que um enxerto de seis fios com grácil duplo extra, de maior espessura, produz um enxerto mais forte.

Como descrito nos artigos acima, diferentes enxertos geram resultados semelhantes. A escolha depende da preferência do cirurgião. Nos joelhos com lesões periféricas, optamos pelos isquiotibiais para reconstrução e enxertos para o mecanismo extensor do pivô central.

Moatshe et al. 28 conduziram um estudo biomecânico em 2018 e observaram melhores resultados tensionando primeiro o LCP, depois o LCA e, então, o canto posterolateral. Franciozi et al. 29 utilizaram um protocolo de tensionamento simultâneo na LB. Estes autores notaram melhor orientação tibiofemoral no grupo em que a fixação do LCA foi feita primeiro. Com base em nossas experiências e de outros autores 21 22 30 preferimos primeiro fixar o LCP em 70 graus de flexão, mantendo o joelho reduzido, e depois o LCA com 20 graus de flexão.

Em um estudo retrospectivo de reconstrução aberta completa em estágio único de lesões ligamentares complexas do joelho, Hirschmann et al. 31 constataram que 79% dos pacientes conseguiram retornar à prática esportiva anterior, mas apenas 33% atingiram o mesmo nível de atividade esportiva anterior à lesão. Acreditamos que os achados diferentes do nosso estudo se devam ao menor nível dos atletas avaliados, assumindo que os atletas de elite aderem a um protocolo de reabilitação mais intensivo.

Não houve infecção. Dois pacientes tiveram artrofibrose e foram submetidos à manipulação 3 meses após a cirurgia. Um paciente apresentou trombose apesar do uso de anticoagulante e foi tratado com dose maior, com boa evolução.

As limitações deste estudo foram o pequeno número de pacientes, a ausência de um grupo controle e a falta de um segundo observador para avaliação dos resultados.

## Conclusão

A reconstrução de LCA e LCP com autoenxerto bilateral dos isquiotibiais é uma opção valiosa para alcance de bons resultados funcionais e estabilidade articular.
